# Untying the Knot: Frenectomy of Lip and Tongue Ties Using Diode Lasers

**DOI:** 10.7759/cureus.76876

**Published:** 2025-01-03

**Authors:** Gauri Kalra, Madhulika Srivastava, Tanu Nangia, Twinkle Chawla

**Affiliations:** 1 Pediatric Dentistry, Manav Rachna Dental College, Faridabad, IND; 2 Pediatric and Preventive Dentistry, Manav Rachna Dental College, Faridabad, IND

**Keywords:** diode laser, frenectomy, frenum, labial, lingual, midline diastema, paediatric dentistry

## Abstract

Frenulum attachments in the oral cavity are pivotal, and any abnormality in their position leads to various issues such as displacement of teeth causing diastema, improper functioning of the tongue affecting deglutition, speech, gingival recession, esthetics, and overall growth of oral musculature and alveolar ridges. Timely diagnosis and correction of frenums is essential following a conservative approach. Diode lasers have been widely used for multiple tissue procedures in dentistry. Due to its superior properties of precise cutting with a blood-free surgical site, minimal pain and discomfort, and minimal need of local anesthesia, laser-assisted frenectomy has been proved to be an efficacious treatment modality in pediatric patients, alleviating stress and anxiety for both children and their caregivers. Thus, a case report of pediatric patients with grade II superior labial frenum (SLF) attachment and class II ankyloglossia treated with diode lasers is presented.

## Introduction

Frenums are muscular attachments in the oral cavity consisting of elastic and collagen fibers as a part of connective tissue. These hypertrophic masses are present since birth and appear to recede as the alveolar bone gains more height during the deposition of bone. They play an essential role in restricting and stabilizing various labial and lingual functional movements in the oral cavity [1]. The labial frenum is often responsible for the displacement of teeth, creating a midline diastema, which gets closed gradually as the frenum recedes with the eruption of permanent anterior teeth [2]. However, any abnormality in these prime structures may hinder various functions such as speech, occlusion, esthetics, functioning of the tongue, gingival recession, and overall growth and development of the mouth. Persistent midline diastema due to high frenum attachment may also lead to ill-fitting prosthesis and plaque accumulation, causing gingival and periodontal diseases [3]. The lingual frenum, a thin tissue fold, too holds significance as it attaches the lingual surface of the tongue to the base of the oral cavity, regulating many vital functions, namely speech and deglutition, and difficulty in latching tongue for breastfeeding in infants. Its abnormal positioning at the tongue’s ventral surface can lead to tongue tie/ankyloglossia, which may limit these functions, compromising overall periodontal health and occlusion [4].

Early diagnosis of abnormally present frenal attachments and their correction following a conservative treatment approach is imperative. High maxillary labial frenums may be identified clinically by carrying out a blanch test and visual examination. Thicker or larger frenums may cover the zone of the attached gingiva and cause changes in the interdental papilla. In the case of lingual attachment, the degree of ankyloglossia may be diagnosed by clinical inspection determining the length of free tongue available for movement and function. Since not only one anatomical type of frenum is solely associated with abnormal tongue functioning, there are grading systems describing the appearance of the lingual frenum that may not categorize the lingual frenum as ankyloglossia. 

Recently, with the advent of soft tissue diode lasers (light amplification by stimulated emission of radiation), frenectomy procedures have become more favorable and patient-compliant. The application of diode lasers in frenectomy has several advantages, such as minimal bleeding and pain, early postoperative healing with minimal inflammation, reduced need for local anesthesia, and a completely sterile working environment. This technology has proved to be a boon for treating pediatric children or patients with exceptional dental healthcare needs. The apparatus is comfortable with a pen-like laser cutting device, which is not frightening for the pediatric patient and provides effective dental treatment, instilling a positive dental attitude among the younger population. This case series highlights two cases of treatment of high maxillary labial frenum and lingual frenum with a soft tissue diode laser in pediatric patients.

## Case presentation

Case of labial frenectomy

**Figure 1:**
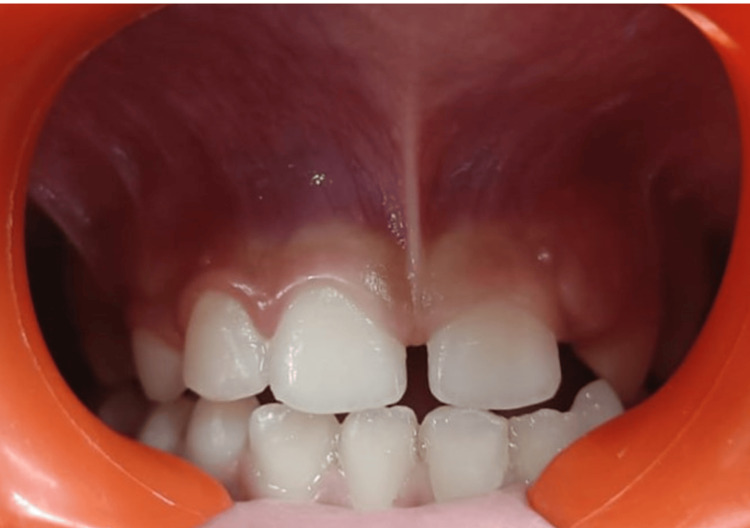
Preoperative clinical view showing high frenal attachment and midline diastema ≈ 3 millimeter (mm)

**Figure 2:**
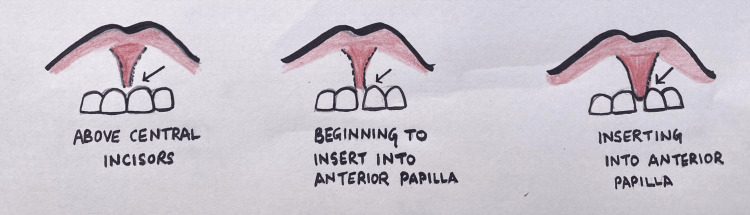
Kotlow's classification of maxillary lip attachments [5]

An eight-year-old male patient reported to the Department of Pediatric and Preventive Dentistry with a chief complaint of space between his maxillary central incisors. The diastema was not present in the primary dentition, raising parental concern. The family reported consulting a dentist earlier, who recommended a wait-and-watch approach during the eruption of the permanent teeth. During the extraoral examination, the patient had a convex profile. The patient had a mixed dentition period with mild maxillary central incisors and bilateral Angle's class I molar relation. Intraoral clinical examination revealed a high labial frenum, 13.5 millimeters (mm) class II, inserted just above and between central incisors (Kotlow’s classification of lip tie, 2013) [5] (Figure 1), causing tension and restriction and a midline diastema of 2.5 mm (Figure 2).

The medical and dental history of the child was insignificant. After evaluating the patient’s history and taking complete oral examination into account, parents were explained about the condition, and frenectomy with diode laser was selected as the treatment of choice. Informed consent was obtained from the parents, and the verbal assent was obtained from the patient before the procedure.

**Figure 3:**
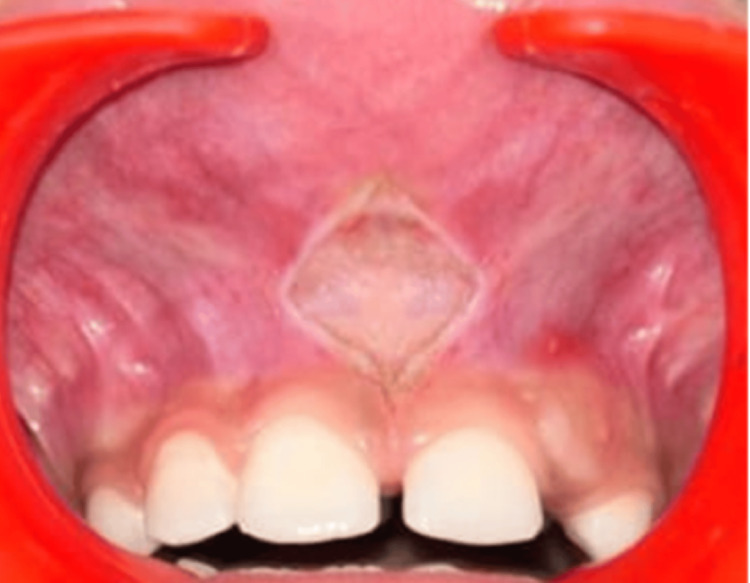
Diamond shaped incision given with the diode laser

The frenectomy was performed using a soft tissue diode laser (LX 16 Plus Dental Diode Laser, Guilin Woodpecker Medical Instrument Co. Ltd., China) with a wavelength of 940 nanometers (nm), operated at 2.0 watts in continuous wave mode, utilizing a 400 micrometer (µm) optical fiber. Topical anesthesia (lidocaine spray) was applied. The laser fiber was initially positioned vertically and laterally, disrupting the continuity of the mucosa. This allowed deeper incisions horizontally in a rhomboidal design (Figure 3).

No sutures were needed to achieve hemostasis, and the patient reported no pain either intraoperatively or postoperatively.

**Figure 4:**
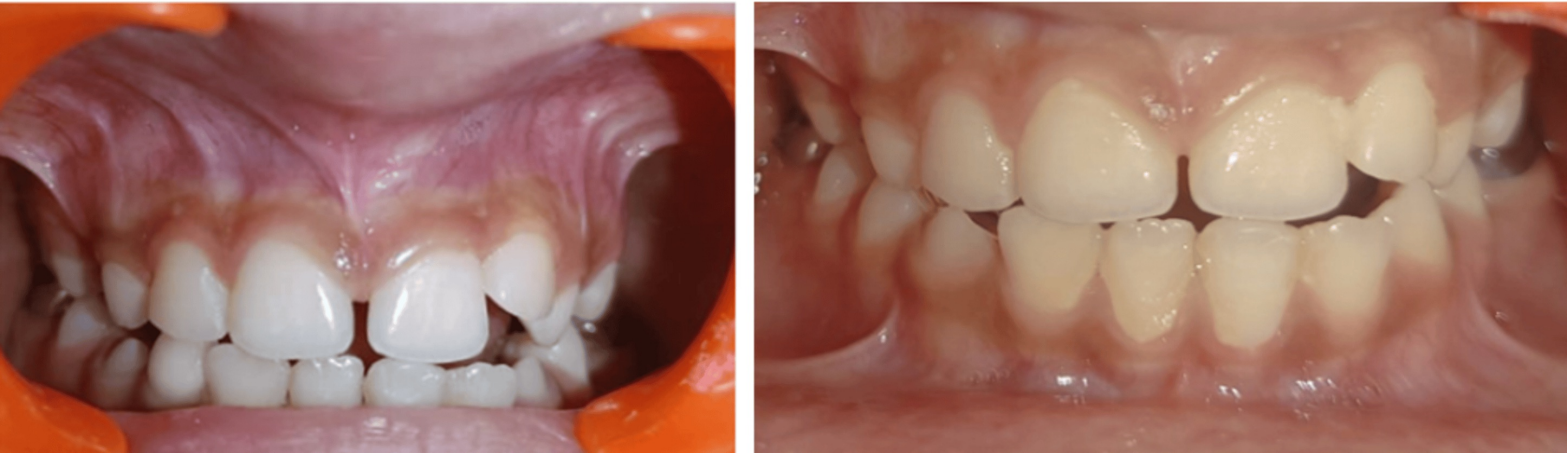
Postoperative clinical follow up view at one week interval and at three months (diastema ≈ 1mm)

The postoperative period was uneventful. There were no complications during or after the procedure, and no analgesics were required. A one-week follow-up revealed complete healing of the surgical site and new frenum insertion. After healing, the beginning of closure of the diastema could be appreciated after one month of follow-up to 2 millimeters (mm). At three months follow-up, further closure of midline diastema to up to 1 mm can be observed after the eruption of maxillary lateral incisors (Figure 4). The patient is under follow-up.

Case of lingual frenectomy

**Figure 5:**
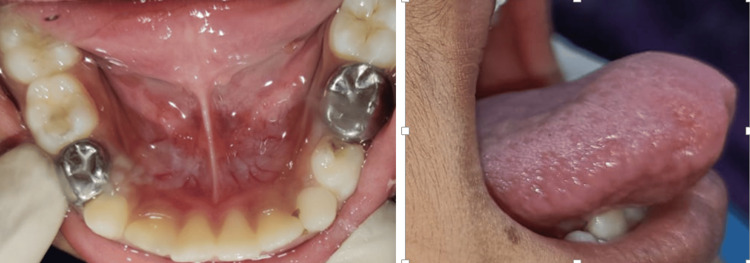
Preoperative clinical frontal view and lateral view showing restricted tongue attachment to floor of the oral cavity

**Figure 6:**
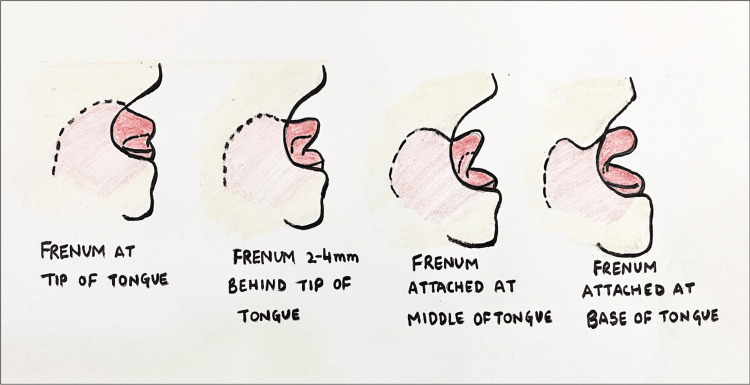
Coryllos’s classification of lingual frenulum [6]

A seven-year-old male presented to the Department of Pediatric and Preventive Dentistry with a chief complaint of pain in the mandibular right posterior region and difficulty pronouncing certain words like 'sh', 't', 'd', 'z' and 'ch'. Clinical examination revealed deep caries in the mandibular left and right posterior teeth, which were determined to be the cause of the pain. Additionally, the patient exhibited signs of ankyloglossia (Coryllos grade II), characterized by an inability to place the tongue on the cingulum of the maxillary incisors and limited tongue protrusion [6] (Figures 5, 6). After a comprehensive evaluation, a treatment plan was formulated, including endodontic therapy for pain management and lingual frenectomy for the correction of ankyloglossia.

**Figure 7:**
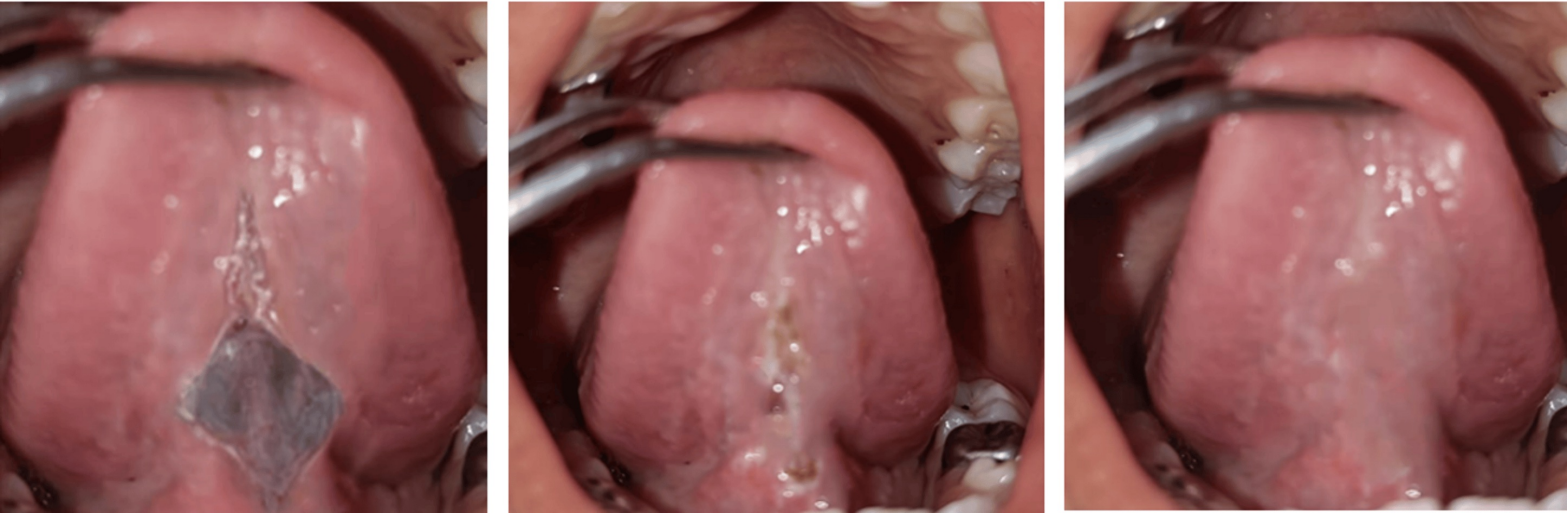
Postoperative clinical view immediately, at one week interval and after one month follow up visit

Following informed consent, a soft tissue diode laser (LX 16 Plus Dental Diode Laser, Guilin Woodpecker Medical Instrument Co. Ltd., China) (wavelength 940 nm) lingual frenectomy was planned. Local anesthesia (2% lignocaine with adrenaline, 1 in 80,000, Xicaine, ICPA Health Products LTD.) was administered with 0.6 milliliter (ml) of lignocaine injected into the frenulum area. A diode laser with a maximum optical power of 2 watts (W), using a 400 µm-initiated fiber optic tip in pulse mode (pulse length: 100 microseconds (µsec), pulse interval: 200 µsec), delivering an average power of 1.3 W, was employed for the procedure. The lingual frenulum was held under tension, and the diode laser was used in contact mode with a focused beam to excise the tissue. The laser tip was moved from the apex to the base of the frenulum using a brushing technique to gradually excise the tissue while continuously mopping with wet gauze to minimize thermal damage. A diamond-shaped wound was observed post-excision, and tongue protrusion was evaluated immediately. No bleeding was noted, and sutures were not required. Follow-up visits were scheduled for one week and one month after the procedure. Healing was uneventful, with significant improvement in tongue mobility observed. The patient demonstrated better tongue protrusion, and the associated speech difficulty improved. Subsequently, the patient was referred to a speech therapist for further speech correction (Figure 7).

## Discussion

Evidence suggests that most dental issues arise with the maxillary labial frenulum and the lingual frenum of varying degrees. Midline diastema and delayed eruption of anterior incisors are commonly associated with the high labial frenulum, and dental anomalies such as speech difficulty, abnormal chewing movements, and deglutition are significant ankyloglossia sequelae. Various classifications of abnormal lip and tongue attachments are present. For the superior labial frenulum (SLF), Kotlow's classification scale is the most commonly applied, including four frenulum types (Kotlow grades I‐IV) based upon the insertion point of the upper frenum onto the gingiva. Kotlow's grade I is considered least restrictive; grade II describes an SLF insertion just above or between the gingival margins of the central incisors; grade III includes an SLF getting inserted into the anterior papilla, and grade IV consists of an SLF inserting into the anterior papilla or wrapping onto the maxillary palatal area [5].

According to Coryllos's classification of lingual frenulum, class 1 is defined as frenum insertion at the tip of the tongue, class 2 is when the lingual frenum is inserted 2-4 mm behind the tip of the tongue, class 3 is defined when the frenum attaches itself to the middle of the tongue, the frenum is thick and less elastic, and class 4 includes a dense, highly inelastic frenulum, which is connected to the base of the tongue [6].

In the present case of labial frenectomy, SLF was inserted on the attached gingiva of the maxilla in between the two incisors (class II), and the lingual frenum was inserted about 2-4 mm behind the tip of the tongue (class II), thus suggesting frenectomy as the treatment of choice.

The ugly duckling stage is a self-correcting anomaly, a characteristic feature of the mixed dentition period [7]. In the first case, the child with high frenal attachment had a mixed dentition period with erupting lateral incisors. However, midline diastema closure was not planned for this patient following the frenectomy, and the patient was kept under a wait-and-watch period till the eruption of incisors. Favorable results have been achieved by reducing the gap between upper incisors. A similar case was reported where midline diastema was immediately corrected within two months after frenectomy without requiring orthodontic intervention [8]. In the second case of a lingual frenectomy, the release of the tongue tie was followed by an intervention by a speech therapist. The role of the speech therapist becomes imperative as it helps to reposition the tongue, helps address any lingering speech issues, improves deglutition, and improves the oral cavity's muscle tone [9,10].

With the advent of minimally invasive dentistry, laser-assisted frenectomy has gained popularity. There are multiple types of hard and soft tissue lasers available these days that can be effectively used for surgical procedures in pediatric patients, such as CO2, erbium (Er):yttrium aluminum garnet (YAG), neodymium-doped YAG (Nd:YAG) with higher wavelengths, and low-wavelength diode lasers. However, a diode laser is exclusively a soft tissue laser and is cost-effective. Because of its small cutting tip, which provides precise cutting, a fiber-optic delivery system, and its selective affinity towards soft tissue pigments, it has proved to be more efficacious than scalpel surgery [11]. It provides a bloodless, sterile surgical site with reduced postoperative pain and inflammation and minimal thermal harm to the soft tissues, providing greater patient acceptance, including anxious pediatric patients, which is comparable to other laser systems [12,13]. A study conducted by Aldelaimi et al. found that out of 25 patients undergoing laser lingual frenectomy, only two complained of postoperative pain and edema and were prescribed analgesics [14]. Thus, with lesser harm to oral tissues and reduced inflammatory responses and postoperative pain, healing had been stated to be better than conventional surgical techniques. Lebret C et al. (2021) conducted a systematic review collating data on perioperative outcomes using diode lasers and conventional surgical methods and concluded achieving satisfactory healing and patient acceptance with laser therapy [3]. 

Future implications

Based on minimally invasive dentistry, laser-assisted procedures are advantageous in pediatric dentistry, providing the best cutting efficacy and minimal discomfort and pain. Laser frenectomies could prevent long-term problems like speech impediments, feeding difficulties, and oral hygiene challenges. Combining recent digital technology and artificial intelligence-guided laser-assisted surgical procedures could provide real-time feedback and optimized tissue cutting, leading to even more precise outcomes.

## Conclusions

The laser-assisted frenectomy procedure for labial, as well as lingual, frenum correction has been proven to be the most suitable treatment strategy in pediatric patients. It offers tissue cutting with precision, complete hemostasis, minimal need for local/topical anesthesia, a sterile surgical site, and minimal postoperative pain and swelling. Moreover, this minimally invasive treatment modality, providing a stress-free environment, is best suited for treating young patients with fear and anxiety.
